# Red blood cell transfusion ≥ 2 units is a risk factor of long-term survival of infectious endocarditis patients with surgical intervention

**DOI:** 10.1097/MD.0000000000045342

**Published:** 2025-11-07

**Authors:** Jing-Bin Huang, Zhai Huang, Chang-Chao Lu, Zhao-Ke Wen

**Affiliations:** a Department of Cardiothoracic Surgery, The People’s Hospital of Guangxi Zhuang Autonomous Region, and Guangxi Academy of Medical Sciences, Nanning, Guangxi, China; b Intensive Care Unit, The People’s Hospital of Guangxi Zhuang Autonomous Region, and Guangxi Academy of Medical Sciences, Nanning, Guangxi, China.

**Keywords:** infectious endocarditis, long-term results, red blood cell transfusion, surgical intervention

## Abstract

Blood transfusion is common in patients with infectious endocarditis (IE) treated surgically. Limited literature on RBC transfusion outcomes in surgical IE. We aimed to clarify impacts of red blood cell (RBC) transfusion on long-term results of IE patients with surgical intervention. We conducted a retrospective study on the medical records of patients with IE undergoing cardiac surgery from 2006 to 2022 in our hospital. In our investigation, 814 IE patients were enrolled into RBC ≥ 2 units group (n = 305) and RBC < 2 units group (n = 509). There were 305 RBC ≥ 2 units patients (37.5%, 305/814). Compared with RBC < 2 units group, all-time mortality (26.2% vs 10.4%, *P <*.001) significantly increased in RBC ≥ 2 units group. We found vegetation diameter ≥ 10 mm, cardiopulmonary bypass length ≥ 120 minutes, aortic occlusion length ≥ 90 minutes, preoperative aortic regurgitation ≥ 4 cm^2^, postoperative left ventricular end diastolic diameter ≥ 70 mm, ventilation length ≥ 72 hours, ang intensive care unit stay ≥ 3 days to be related to RBC ≥ 2 units. RBC ≥ 2 units is significantly related to 1- and 5-year mortality after cardiac operation and all-time mortality. The presence of RBC ≥ 2 units in IE is a risk factor of long-term survival. In our investigation, the presence of RBC ≥ 2 units has adverse impact on long-term results of IE patients with surgical intervention. The management strategies for IE anemia may not be limited to blood transfusions, but also include drug treatments such as iron supplements and red blood cell stimulants. This study provides valuable information for clinical practice of blood transfusion in cardiac surgery.

## 1. Introduction

Infectious endocarditis (IE) is an infection of the heart valves and inner layers of the heart chamber. IE remains a life-threatening condition with high early and late mortality. The mortality rate within one year is close to 30%, and the global IE incidence rate is still rising. The mortality rate of IE is high worldwide.^[1–4]^ Blood is a scarce resource. More than 100 million units of blood are collected worldwide every year. Infusing red blood cells (RBC) is one of the few management methods that can fully restore tissue oxygenation. According to the AABB guidelines in 2023, hemoglobin level is used for restrictive transfusion threshold, which is lower than 70 g/L in most adult patients, lower than 80 g/L in patients with symptomatic cardiovascular disease, and lower than 75 g/L in patients with cardiac surgery. In cases of acute blood loss, especially uncontrolled hemorrhagic shock, transfusion of red blood cells may save lives. Red blood cell transfusion will continue to be an indispensable part of cardiac anesthesia practice.^[4–8]^

Blood transfusion is common in patients with IE treated surgically. Investigations of the impacts of transfusion of red blood cells on long-term results of IE patients with surgical intervention are rare.^[6,8–11]^ We aimed to analyze the impacts of transfusion of red blood cells on long-term results of IE patients with surgical intervention.

## 2. Materials and methods

We conducted a retrospective study on the medical records of patients with IE undergoing cardiac surgery from 2006 to 2022 in our hospital, aiming to clarify impacts of red blood cell (RBC) transfusion on long-term results of IE patients with surgical intervention. The diagnosis is based on the modified Duke criteria. The RBC units were transfused both intraoperatively and postoperatively.

### 2.1. Variable

We conducted an investigation on the parameters (see Supplementary Chapter for data, Supplemental Digital Content, https://links.lww.com/MD/Q446).

Patients with missing data, prior operations were excluded from the study.

### 2.2. Follow-up

From discharge to death or the end of the study, all patients will undergo echocardiography, electrocardiography, and chest X-ray examinations every 3 to 12 months. At the last follow-up, the patient was interviewed at the outpatient department or contacted via phone or WeChat. The data of routine X-ray, ECG, and echocardiographic monitoring of patients discharged were obtained from the out-patient record system. We used multiple imputation to fill these missing follow-up data.

### 2.3. Statistical analyses

We analyzed continuous variables using Wilcoxon rank sum test and reported them as median and interquartile range. We also presented categorical data in the form of frequency and percentage, and investigated it using chi square test or Fisher exact test. We used logistic regression and created Kaplan–Meier curves and compared them using logarithmic rank test. Association among 2 quantities was determined by the Spearman correlation coefficient. Risk factors were evaluated by logistic regression analysis. The receiver operating characteristic (ROC) curve with the respective area under the curves was plotted to investigate the diagnostic value of the risk. The optimal cutoff was assessed by using Youden index in ROC analysis. All tests are bilateral, and statistical significance is defined by a *P*-value < .05. We used IBM SPSS 24.0 software (IBM SPSS Inc., Armonk) to complete the analysis.

## 3. Results

### 3.1. Characteristics of the population with infectious endocarditis

**Figure 1.**
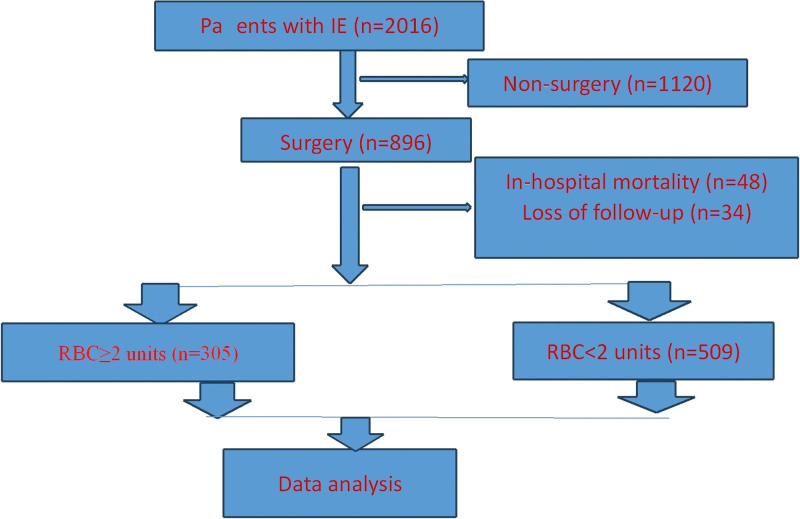
Flow chart of patients.

During the study period, 2016 patients were diagnosed as infective endocarditis, 1120 cases were non-surgical, 896 cases were surgical, 48 cases died in hospital, 34 cases were lost to follow-up. 814 IE patients who were successfully followed up were included in this study and enrolled into RBC ≥ 2 units group (n = 305) and RBC < 2 units group (n = 509) (Fig. 1). There were 305 RBC ≥ 2 units patients (37.5%, 305/814) (Table 1).

**Table 1:** Preoperative file, surgical and follow-up results.

Variable	RBC ≥ 2 units (n = 305)	RBC < 2 units (n = 509)	*P*-value
Preoperative			
Male gender, n (%)	214 (70.2%)	326 (64.0%)	.074
Age, yr	45.04 ± 14.01	34.38 ± 13.47	<.001
Weight, kg	58.10 ± 11.78	54.69 ± 12.20	<.001
Duration between symptoms and operation, mo	3.50 ± 3.53	2.08 ± 1.63	<.001
Vegetation size, mm	12.95 ± 6.85	8.05 ± 5.88	<.001
Preoperative LVEDD, mm	64.36 ± 8.98	58.91 ± 9.20	<.001
Preoperative LVEF, %	58.89 ± 9.0	61.88 ± 7.42	<.001
Preoperative aortic regurgitation, cm^2^	6.87 ± 5.54	4.65 ± 7.18	<.001
Preoperative mitral regurgitation, cm^2^	7.77 ± 6.20	6.91 ± 6.05	.054
Preoperative tricuspid regurgitation, cm^2^	3.73 ± 3.20	5.31 ± 5.50	<.001
Serum creatinine before surgery, μmol/L	88.96 ± 20.90	69.67 ± 21.03	<.001
Operative			
CPB time, min	161.30 ± 45.56	123.52 ± 41.24	<.001
Mechanical ventilation time, h	57.84 ± 47.86	28.45 ± 36.66	<.001
Length of ICU stay, d	6.12 ± 2.88	3.56 ± 2.14	<.001
Postoperative hospital stay, d	20.86 ± 8.61	18.14 ± 6.17	<.001
Creatinine of serum 24 h after surgery, μmol/L	106.30 ± 34.50	74.90 ± 33.61	<.001
Creatinine of serum 48 h after surgery, μmol/L	132.31 ± 58.78	73.74 ± 36.70	<.001
Chest drainage, mL	796.49 ± 340.16	513.24 ± 381.03	<.001
Postoperative LVEDD, mm	48.64 ± 7.08	46.86 ± 7.11	<.001
Postoperative LVEF, %	58.89 ± 9.0	61.88 ± 7.42	<.001
Frozen plasma transfusion, mL	911.64 ± 399.90	438.19 ± 414.83	<.001
Follow-up			
Length of follow-up, mo	65.13 ± 55.27	71.78 ± 50.54	<.001
All-time mortality, n	80 (26.2%)	53 (10.4%)	<.001

CPB = cardiopulmonary bypass, ICU = intensive care unit, LVEDD = left ventricular end diastolic diameter, LVEF = left ventricular ejection fractions, RBC = red blood cells.

### 3.2. Comparison between RBC ≥ 2 and <2 units groups

Age (45.04 ± 14.01 vs 34.38 ± 13.47 years, *P* < .001), body weight (58.10 ± 11.78 vs 54.69 ± 12.20 kg, *P* < .001), duration between symptoms and operation (3.50 ± 3.53 vs 2.08 ± 1.63 months, *P* < .001), vegetation size (12.95 ± 6.85 vs 8.05 ± 5.88 mm, *P* < .001), preoperative left ventricular end diastolic diameter (LVEDD) (64.36 ± 8.98 vs 58.91 ± 9.20 mm, *P* < .001), preoperative aortic regurgitation (6.87 ± 5.54 vs 4.65 ± 7.18 cm^2^, *P* < .001), and serum creatinine before surgery (88.96 ± 20.90 vs 69.67 ± 21.03 μmol/L, *P* < .001) in RBC ≥ 2 units group were significantly higher than those in RBC < 2 units group (Table 1). Preoperative left ventricular ejection fractions (LVEF; 58.89 ± 9.0 vs 61.88 ± 7.42 %, *P* < .001) and preoperative tricuspid regurgitation (3.73 ± 3.20 vs 5.31 ± 5.50 cm^2^, *P* < .001) in RBC ≥ 2 units group were significantly less than both in RBC < 2 units group (Table 1).

Cardiopulmonary bypass (CPB) time (161.30 ± 45.56 vs 123.52 ± 41.24 minutes, *P* < .001), mechanical ventilation time (57.84 ± 47.86 vs 28.45 ± 36.66 hours, *P* < .001), length of intensive care unit (ICU) stay (6.12 ± 2.88 vs 3.56 ± 2.14 days, *P* < .001), postoperative hospital stay (20.86 ± 8.61versus 18.14 ± 6.17 days, *P* < .001), serum creatinine 48 hours after surgery (132.31 ± 58.78 vs 73.74 ± 36.70 μmol/L, *P* < .001), postoperative LVEDD (48.64 ± 7.08 vs 46.86 ± 7.11 mm, *P* < .001), chest drainage (796.49 ± 340.16 vs 513.24 ± 381.03 mL, *P* < .001), fresh-frozen plasma (911.64 ± 399.90 vs 438.19 ± 414.83 mL, *P* < .001), vegetation size ≥ 10 mm (53.3% vs 43.2%, *P* < .001), surgical patients (74.7% vs 21.3%, *P* < .001), and time between symptoms and admission ≥ 2 months (51.1% vs 44.4%, *P* = .004) in RBC ≥ 2 units group were significantly higher than those in RBC < 2 units group (Table 1). Postoperative LVEF (58.89 ± 9.0 vs 61.88 ± 7.42 %, *P* < .001) in RBC ≥ 2 units group was significantly less than that RBC < 2 units group (Table 1).

### 3.3. Follow-up data

The average follow-up time was 75.14 ± 1.80 months (range, 1–204). 87 cases (87/814, 10.7%) died within 12 months of discharge due to IE recurrence and cerebral hemorrhage. The latest follow-up data shows that 681 survivors belong to New York Heart Association class I (85.0%), and 109 survivors belong to class II (15.0%). Length of follow-up (65.13 ± 55.27 vs 71.78 ± 50.54 months, *P* < .001) in RBC ≥ 2 units group was statistically significantly less than that in RBC < 2 units group. Compared with RBC < 2 units group, all-time mortality (26.2% vs 10.4%, *P* < .001) significantly increased in RBC ≥ 2 units group (Table 1).

Operation and causes of in-hospital mortality and complications in IE are shown Table 2.

**Table 2:** Operation and causes of in-hospital mortality and complications in infectious endocarditis (n = 896).

Variable	Value	Mortality
Operation		
In-hospital mortality		5.36% (48/896)
AVR isolated, %	19.64% (176/896)	1.34% (12/896)
MVR isolated, %	41.07% (368/896)	1.79% (16/896)
Double valve operation, %	28.57% (256/896)	2.23% (20/896)
Bentall + MVR, %	1.79% (16/896)	0
Tricuspid annuloplasty isolated, %	8.92% (80/896)	0
ECMO, %	0.33% (3/896)	
Causes of in-hospital mortality	%	
Paravalvular leak + septicemia + AKI + hepatic failure + cardiogenic shock	3.57% (32/896)	
Encephalorrhagia	1.79% (16/896)	
Complications		
Mechanical ventilation time >72 h, %	21.43% (192/896)	
Respiratory failure, %	15.07% (135/896)	
Acute kidney injury, %	28.68% (257/896)	
Liver failure, %	4.35% (39/896)	
Ventricular fibrillation, %	3.68% (33/896)	

AKI = acute kidney injury, AVR = aortic valve replacement, ECMO = extracorporeal membrane oxygenation, MVR = mitral valve replacement.

The early postoperative complications were mechanical ventilation time > 24 hours (39.3%), acute kidney injury (30.4%) and multiple organ failure (9.6%).

### 3.4. Risk factors of RBC ≥ 2 units

By univariate analysis, vegetation diameter ≥ 10 mm (OR: 3.458, 95% CI: 2.569–4.654, *P* < .001), CPB length ≥ 120 minutes (OR: 5.629, 95% CI: 4.036–7.851, *P* < .001), aortic occlusion length ≥ 90 minutes (OR: 8.244, 95% CI: 5.952–11.419, *P* < .001), preoperative aortic regurgitation ≥ 4 cm^2^ (OR: 2.949, 95% CI: 2.194–3.692, *P* < .001), postoperative LVEDD ≥ 70 mm (OR: 4.454, 95% CI: 3.010–6.589, *P* < .001), ventilation length ≥ 72 hours (OR: 4.636, 95% CI: 3.127–6.873, *P* < .001), ang ICU stay ≥ 3 days (OR: 3.072, 95% CI: 2.277–4.145, *P* < .001) were showed to be related to RBC ≥ 2 units. (Table 3)

**Table 3:** Predictor of RBC ≥ 2 units.

Model	OR	95% CI	*P*-value
Univariate analysis			
Vegetation diameter ≥ 10 mm	3.458	2.569–4.654	<.001
CPB length ≥ 120 min	5.629	4.036–7.851	<.001
Aortic occlusion length ≥ 90 min	8.244	5.952–11.419	<.001
Preoperative aortic regurgitation ≥ 4 cm^2^	2.949	2.194–3.692	<.001
Postoperative LVEDD ≥ 70 mm	4.454	3.010–6.589	<.001
Ventilation length ≥ 72 h	4.636	3.127–6.873	<.001
ICU stay ≥ 3 d	3.072	2.277–4.145	<.001
Multivariate analysis			
Vegetation diameter ≥ 10 mm	3.905	2.809–5.430	<.001
CPB length ≥ 120 min	5.170	3.543–7.563	<.001
Aortic occlusion length ≥ 90 min	6.449	3.548–11.721	<.001
Preoperative aortic regurgitation ≥ 4 cm^2^	3.091	2.138–4.468	<.001
Postoperative LVEDD ≥ 70 mm	5.286	3.315–8.430	<.001
Ventilation length ≥ 72 h	4.117	2.703–6.272	<.001
ICU stay ≥ 3 d	3.700	2.645–5.177	<.001

CPB = cardiopulmonary bypass, ICU = intensive care unit, LVEDD = left ventricular end diastolic diameter, RBC = red blood cells.

By multivariate analyses, vegetation diameter ≥ 10 mm (OR: 3.905, 95% CI: 2.809–5.430, *P* < .001), CPB length ≥ 120 minutes (OR: 5.170, 95% CI: 3.543–7.563, *P* < .001), aortic occlusion length ≥ 90 minutes (OR: 6.449, 95% CI: 3.548–11.721, *P* < .001), preoperative aortic regurgitation ≥ 4 cm^2^ (OR: 3.091, 95% CI: 2.138–4.468, *P* < .001), postoperative LVEDD ≥ 70 mm (OR: 5.286, 95% CI: 3.315–8.430, *P* < .001), ventilation length ≥ 72 hours (OR: 4.117, 95% CI: 2.703–6.272, *P* < .001), ang ICU stay ≥ 3 days (OR: 3.700, 95% CI: 2.645–5.177, *P* < .001) were showed to be related to RBC ≥ 2 units (Table 3).

### 3.5. Implications of RBC ≥ 2 units

Univariate analysis indicated that RBC ≥ 2 units is statistically significantly related to 1-year mortality after heart surgery (OR: 1.827, 95% CI: 1.111–3.004, *P* = .018) and 5-year mortality after cardiac operation (OR: 2.060, 95% CI: 1.500–2.828, *P* < .001) respectively (Table 4).

**Table 4:** Implication of RBC ≥ 2 units (n = 814).

Model	OR	95% CI	*P*-value
Univariate analysis of risk factors of 1-yr mortality after cardiac operation (n = 87)			
RBC ≥ 2 units	1.827	1.111–3.004	.018
Multivariate analysis of risk factors of 1-yr mortality after cardiac operation (n = 87)			
RBC ≥ 2 units	1.939	1.138–3.305	.015
Univariate analysis of risk factors of 5-yr mortality after cardiac operation (n = 100)			
RBC ≥ 2 units	2.060	1.500–2.828	<.001
Multivariate analysis of risk factors of 5-yr mortality after cardiac operation (n = 100)			
RBC ≥ 2 units	2.171	1.286–3.663	.004

RBC = red blood cells.

Multivariate analysis indicated that indicated that RBC ≥ 2 units is statistically significantly related to 1-year mortality after cardiac operation (OR: 1.939, 95% CI: 1.138–3.305, *P* = .015) and 5-year mortality after cardiac operation (OR: 2.171, 95% CI: 1.286–3.663, *P* = .004) respectively (Table 4).

### 3.6. Cox proportional risk regression of all-time mortality in follow-up

Univariate analysis of Cox proportional hazard regression for all-time mortality identified CPB time ≥ 180 minutes (HR: 5.686, 95% CI: 4.005–8.072, *P* < .001), Aortic occlusion length ≥ 90 minutes (HR: 7.309, 95% CI: 4.583–11.656, *P* < .001) and RBC ≥ 2 units (HR: 2.648, 95% CI: 1.869–3.752, *P* < .001) to be associated with all-time mortality in follow-up (Table 5).

**Table 5:** Cox proportional risk regression of all-time mortality in follow-up (n = 814).

Model	HR	95% CI	*P*-value
Univariate analysis			
CPB time ≥ 180 min	5.686	4.005–8.072	<.001
Aortic occlusion length ≥ 90 min	7.309	4.583–11.656	<.001
ICU stay ≥ 3 d	8.056	4.779–13.612	<.001
RBC ≥ 2 units	2.648	1.869–3.752	<.001
Multivariate analysis			
CPB time ≥ 180 min	2.508	1.350–4.660	<.001
Aortic occlusion length ≥ 90 min	7.626	4.681–12.424	<.001
ICU stay ≥ 3 d	8.821	5.189–14.995	<.001
RBC ≥ 2 units	3.362	1.615–3.454	<.001

CPB = cardiopulmonary bypass, ICU = intensive care unit, RBC = red blood cells.

Multivariate analysis of Cox proportional hazard regression for all-time mortality identified CPB time ≥ 180 minutes (HR: 2.508, 95% CI: 1.350–4.660, *P* < .001), aortic occlusion length ≥ 90 minutes (HR: 7.626, 95% CI: 4.681–12.424, *P* < .001) and RBC ≥ 2 units (HR: 3.362, 95% CI: 1.615–3.454, *P* < .001) to be associated with all-time mortality in follow-up (Table 5).

### 3.7. Association between CPB length and RBC  ≥ 2 units

**Figure 2.**
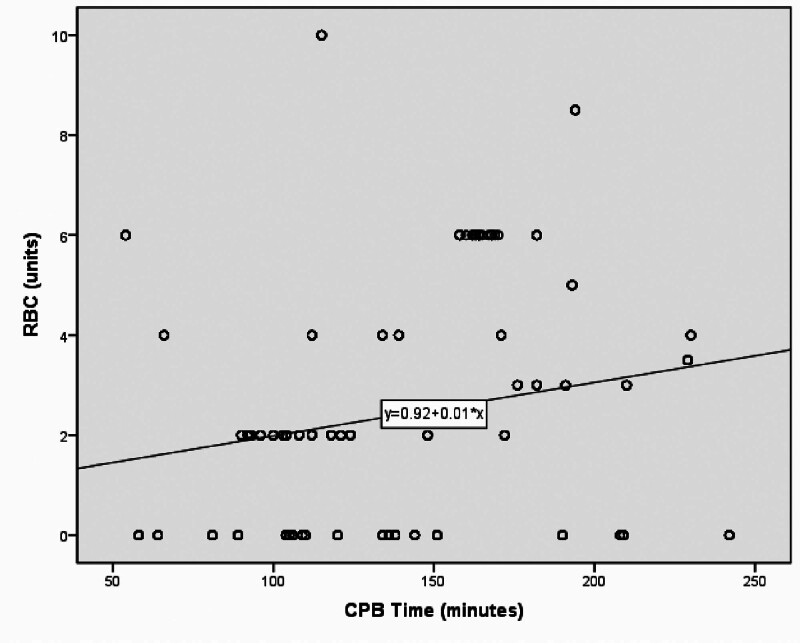
Association between CPB length and RBC ≥ 2 units. Spearman correlations analysis showed a moderate positive correlation between CPB length and RBC ≥ 2 units. CPB = cardiopulmonary bypass, RBC = red blood cells.

Spearman correlations analysis showed a moderate positive correlation between CPB length and RBC ≥ 2 units (*R* = 0.334, *P* < .001) (Fig. 2).

### 3.8. The ROC curve of diagnostic accuracy with CPB length for RBC ≥ 2 units

**Figure 3.**
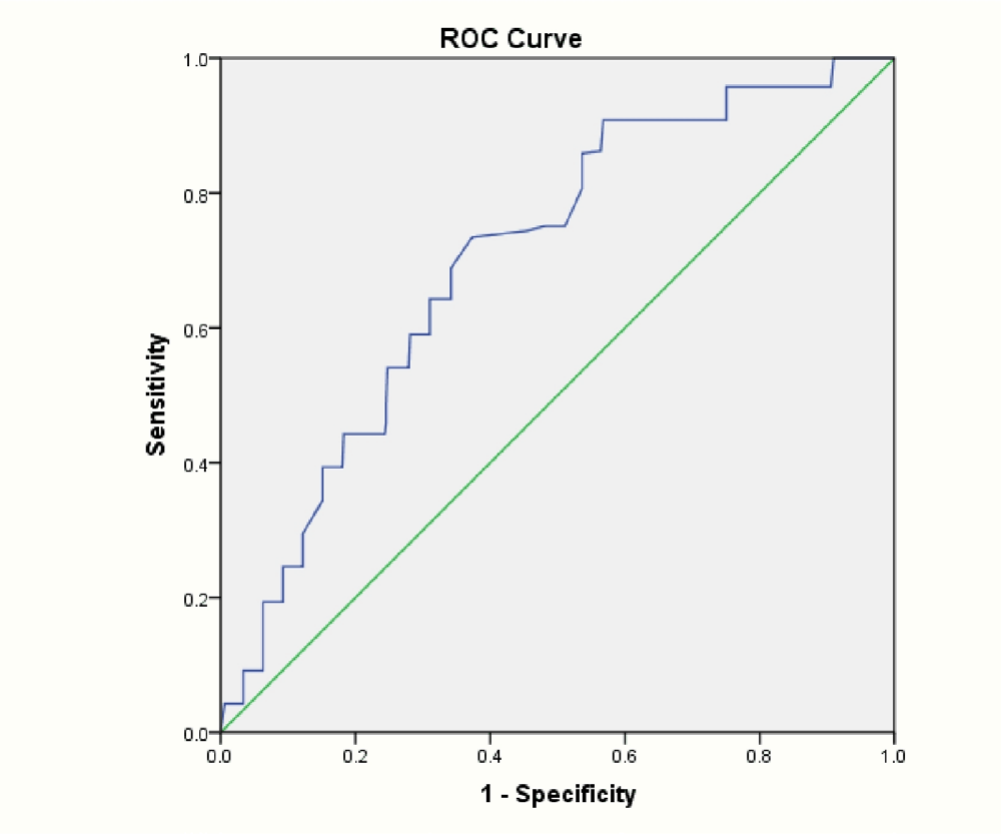
The ROC curve of diagnostic accuracy with CPB length for RBC ≥ 2 units. A value of CPB length > 129 min was 73.4% sensitive and 62.7% specific for the diagnosis of RBC ≥ 2 units, with an AUC 0.704. AUC = area under the curve, CPB = cardiopulmonary bypass, RBC = red blood cells, ROC = receiver operating characteristic.

A value of CPB length > 129 minutes was 73.4% sensitive and 62.7% specific for the diagnosis of RBC ≥ 2 units, with an area under the curves 0.704 (95% confidence interval: 0.668–0.740; *P* < .001) and Youden index 0.361 (Fig. 3).

### 3.9. Follow up

**Figure 4.**
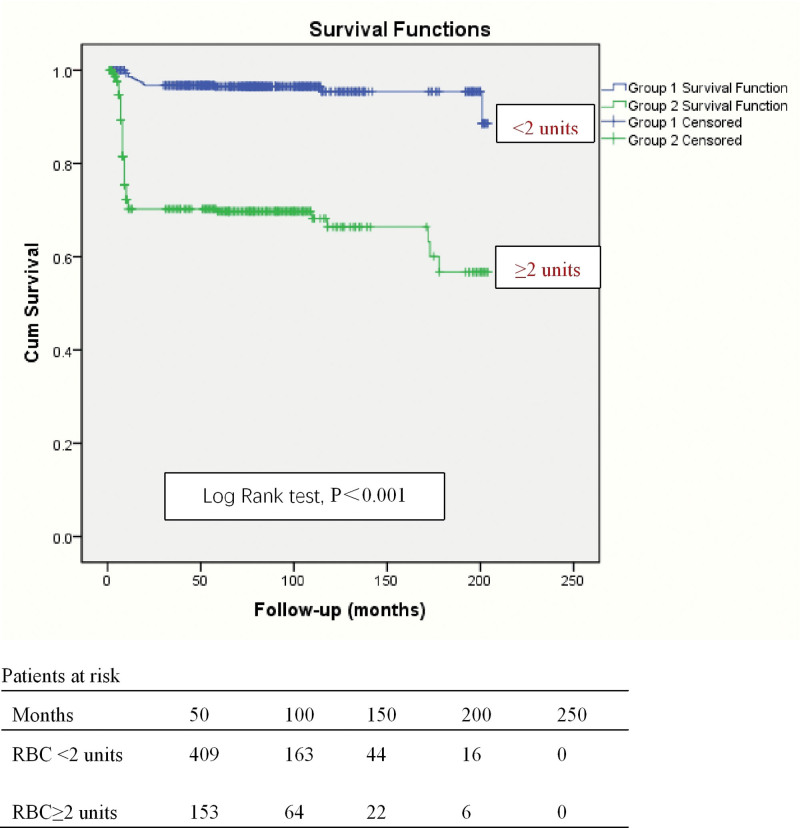
Kaplan–Meier curve for survival. The presence of RBC ≥ 2 units in infectious endocarditis is a risk factor of long-term survival. Blue line, RBC < 2 units; Green line, RBC ≥ 2 units. Cum survival = Cumulative survival, CPB = cardiopulmonary bypass, RBC = red blood cells.

The presence of RBC ≥ 2 units in IE is a risk factor of long-term survival (Log-Rank test, *P* < .001) (Fig. 4).

## 4. Discussion

In our investigation, the incidence rate of patients with IE and surgical intervention with RB C ≥ 2 units transfusion was 37.5%. RBC ≥ 2 units is significantly related to 1-, 5-year and all-time mortality in follow-up. A positive correlation between CPB length and RBC ≥ 2 units exist.

The treatment of IE anemia includes diagnostic evaluation, treatment intervention, and blood transfusion. In this situation, transfusion practice is particularly challenging as it requires balancing the management of anemia with potential risks related to transfusion, including transfusion reactions, immune regulation, and increased risk of infection. In addition, the optimal transfusion threshold and strategy for these patients are unknown.^[12–15]^

Systemic inflammation in IE patients leads to inflammatory anemia, which affects the production and clearance of red blood cells (RBC). IE triggers a systemic inflammatory response characterized by increased inflammatory cytokines including interleukin-6. Subsequently, these circulating inflammatory cytokines achieve anemia through inflammation driven iron restriction, inhibition of red blood cell production, and decreased red blood cell survival rate. Iron restriction is mediated by excessive production of hepcidin and driven by cytokines including interleukin-6.^[16,17]^ Hepcidin blocks iron output by degrading ferritin, capturing iron in macrophages, and reducing dietary iron absorption, resulting in decreased availability of iron produced by red blood cells. In addition, inflammatory cytokines including tumor necrosis factor and interleukin-1 directly inhibit the activity of red blood cell production, further leading to anemia. These cytokines reduce the production of erythropoietin and their signaling, limiting the production of red blood cells, although the body needs to compensate for low hemoglobin levels. The inflammatory damage to erythroid progenitor cells exacerbates this inhibition, leading to a weakened response to erythropoietin.^[18–20]^

Hemolysis or destruction of red blood cells also contribute to IE anemia. Hemolysis can be caused by various mechanisms, such as preexisting valve stenosis, vegetations, artificial heart valves, perivalvular leakage, or immune-mediated red blood cell destruction leading to mechanical damage to red blood cells, as described above.^[21]^

It has been reported that iron deficiency is popular in the general population, and its incidence rate reaches up to 50% in patients with cardiovascular diseases including heart failure and aortic valve disease. The exact percentage of IE patients with iron deficiency anemia is not yet clear. Although it is conceivable that iron deficiency plays a part in i.e. related anemia in some patients, its exact role still needs to be further clarified.^[22,23]^

Malnutrition and the presence of mechanical heart valves are the other influencing factors. Although rare, some antibiotics used to treat IE including beta lactam antibiotics, may cause bone marrow suppression or drug-induced immune hemolytic anemia. Practice of blood transfusion must strike a balance between the supporting oxygen delivery need and transfusion risks. Based on the 2023 AABB guidelines, it is generally recommended to limit the transfusion threshold, with most adult patients having hemoglobin levels below 70 g/L (strongly recommended, moderate certainty evidence), and symptomatic cardiovascular disease patients having hemoglobin levels below 80 g/L, clinical doctors can use a threshold of 75 g/L for patients undergoing heart operation.^[24,25]^

Blood transfusion decisions can also consider the severity of symptoms, clinical background, and comorbidities, especially considering the possibility of systemic inflammation and coagulation disorders in IE. Although the restrictive red blood cell transfusion strategy appears to be safe in most clinical settings, it has been reported that the results of MINT trials suggest that the free transfusion strategy may improve the prognosis of patients with acute myocardial infarction and anemia. Therefore, further research is necessary, especially in patients with specific cardiovascular diseases such as IE. The data on the incidence of blood transfusion in IE patients is limited. In patients receiving conservative treatment, the transfusion rate is 14 to 18.5%. There is a significant difference in the red blood cell infusion rate among patients undergoing IE cardiac surgery, with a research report range of 38 to 88%. This universal transfusion rate is not unique to IE, but reflects a broader pattern observed in general cardiac surgery and ICUs. Differences in transfusion practices between hospitals are at least partly due to differences in institutional culture and established programs. It has been reported that IE is an independent risk factor for allogeneic blood transfusion. Similarly, IE has been identified as a risk factor of intraoperative large blood transfusions (exceeding 4 units of red blood cells), with some patients requiring more than 10 units of red blood cells during surgery. In addition, patients undergoing valve replacement surgery appear to have a greater risk of blood transfusion compared to those undergoing valve repair. Artificial valve IE operation needs more blood transfusions than natural valve IE operation.^[26–29]^ In line with the trend of regular cardiac surgery, female patients receive more red blood cell transfusions during surgical intervention compared to male patients.^[29,30]^

Although literature is limited, many studies have shown that anemia is associated with an increased mortality rate in IE. Studies mainly focused on anemia in IE and showed that as the severity of anemia worsens, the risk of death also increases. Anemia is a risk factor for mortality and has been included in their put forward risk scoring system for predicting mortality after IE surgery. However, subsequent validation studies on various scoring systems, including ANCLA, found no significant correlation between anemia and in-hospital mortality. Nevertheless, the ANCLA scoring system has been proven to be the most accurate in predicting mortality rates in IE patients.^[31–33]^ The negative correlation between anemia and the prognosis of patients with IE is in line in the literature reports. Large prospective and multicenter studies show that there is a clear correlation between preoperative anemia and poor prognosis, such as acute kidney injury, longer mechanical ventilation length, more red blood cell infusion and increased in-hospital mortality.^[34–36]^

Although it is usually necessary, red blood cell transfusion may have negative effects on IE patients. It has been reported a correlation between red blood cell transfusion and adverse outcomes. Other studies have shown a correlation between intraoperative red blood cell transfusion and prolonged ICU stay and acute kidney injury. For IE patients, the risk of renal function deterioration after blood transfusion may be high, as they typically already have impaired renal function. However, it is currently unclear whether blood transfusion itself is the main cause of adverse reactions, or whether potential diseases that require blood transfusion, such as anemia and bleeding, play a more important role.^[36–38]^

Although it is usually necessary, red blood cell transfusion may have negative effects on IE patients. A correlation between red blood cell transfusion and bad clinical results has been found. It has been shown a correlation between intraoperative red blood cell transfusion and delayed ICU duration and acute kidney injury. For IE patients, the risk of worsen renal function after blood transfusion may be high, as they have preoperative impaired renal function.^[36–38]^ Previous studies in heart operation have found a correlation between blood transfusion during heart operation and the risk of acute kidney injury, with the risk being more serious in anemic patients compared to non-anemic patients. The potential pathophysiological mechanisms of surgical injury include preoperative renal hypoxia (anemia and decreased oxygen supply), oxidative stress, inflammatory mediators, and excessive iron and free hemoglobin load caused by blood transfusion.^[35–37]^ In our investigation, RBC ≥ 2 units is significantly related to 1-, 5-year and all-time mortality in follow-up. It has been reported that Long-term survival in the group with AKI was markedly lower than that in the group without AKI (Log-Rank test, *P* = .009), supporting our finding.^[39]^

### 4.1. Management strategies for anemia in infectious endocarditis

There is currently no published data on the effectiveness of drug intervention in the management of i.e. anemia. However, implementing a Patient Blood Management program for IE patients may be a reasonable approach. Typical strategies in Patient Blood Management include oral and intravenous iron supplements, iron supplementation, erythropoietic stimulants, or combinations of these therapies. Oral iron supplementation has become a common treatment for iron deficiency. But it is not sufficient for patients needing rapid and effective preoperative iron supplementation. In this case, the limitations of oral iron supplements include prolonged treatment time, low bioavailability, and poor tolerance for patient to oral iron. Iron supplementation has higher bioavailability and better tolerance.^[40,41]^ A meta-analysis of the impact of iron supplementation in various medical and surgical specialties showed that the group of patients receiving iron supplementation treatment had significantly elevated hemoglobin levels and reduced red blood cell transfusions. In addition, a recent meta-analysis of iron supplementation in cardiac surgery patients showed that iron supplementation can effectively reduce postoperative RBC infusion rate and increase hemoglobin levels, especially on days 4 to 10 and 21. However, iron supplementation does not seem to affect mortality, kidney function, or ICU duration. However, because of a lack of study, the efficacy of iron supplementation in IE patients is still unknown. Iron supplementation is related to an increased risk of infection, for improved free serum iron may increase bacterial growth. A recent meta-analysis on iron supplementation in a mixed population found that the group receiving iron supplementation treatment had an increased risk of infection. Erythropoietic stimulants has not yet been studied in IE patients, although they have been extensively studied in various other clinical settings. A recent meta-analysis showed that erythropoietic stimulants, when combined with iron supplementation, significantly reduces the transfusion needs of patients undergoing cardiac surgery. Interestingly, some studies have shown that shortening erythropoietic stimulants treatment time can achieve promising results. It has been reported that a single dose of erythropoietin 2 days before surgery can effectively reduce perioperative red blood cell transfusion, and improve the hemoglobin level on the fourth day. Even the combination of erythropoietin treatment with iron supplementation, folic acid, and vitamin B12 on the day before surgery seems to lessen transfusion requirements, this positive effect being reflected in all causes of anemia.^[42–44]^

The study of IE related anemia may include adjuvant therapy for the underlying mechanism of anemia. IL-6 inhibitors, such as tocilizumab, have been shown to inhibit hepcidin proliferation and improve anemia in inflammatory autoimmune diseases including rheumatoid arthritis and Castleman disease. The impact of these treatments on patients with IE is unknown. Future studies should clarify the potential benefits and mechanisms of these new drugs.^[40–46]^

## 5. Limitations

The limitation of this study lies in its retrospective design, which may lead to selection bias due to the retrospective nature of the study and our hospital’s role as a tertiary referral center. Long term recruitment of patients may have adverse effects on the accuracy of the results. Prospective randomized controlled trials are needed, and plans to decrease the rate of incidence and mortality of nosocomial IE need to be developed. The results of multivariate logistic regressions are very similar, which increases the possibility of confusion and insufficient adjustment. But the reasons need to be further elaborated. Our study identified that ICU stay and ventilation hours to be predictors of transfusion, these may be consequences of transfusion or surgical complexity.

## 6. Conclusions

In our investigation, the presence of RBC ≥ 2 units has adverse impact on long-term results of IE patients with surgical intervention. The management strategies for IE anemia may not be limited to blood transfusions, but also include drug treatments such as iron supplements and red blood cell stimulants. This study provides valuable information for clinical practice of blood transfusion in cardiac surgery.

## Author contributions

**Conceptualization:** Jing-Bin Huang.

**Funding acquisition:** Jing-Bin Huang.

**Investigation:** Jing-Bin Huang, Chang-Chao Lu, Zhao-Ke Wen.

**Methodology:** Jing-Bin Huang.

**Resources:** Jing-Bin Huang, Zhai Huang, Chang-Chao Lu.

**Supervision:** Zhao-Ke Wen.

**Validation:** Zhai Huang, Chang-Chao Lu, Zhao-Ke Wen.

**Visualization:** Zhai Huang, Chang-Chao Lu.

## Supplementary Material


